# Assessment of Factors Influencing Morale in the Elderly

**DOI:** 10.1371/journal.pone.0016490

**Published:** 2011-01-25

**Authors:** Seng Cheong Loke, Siti S. Abdullah, Sen Tyng Chai, Tengku A. Hamid, Nurizan Yahaya

**Affiliations:** 1 Institute of Gerontology, Universiti Putra Malaysia, Serdang, Selangor Darul Ehsan, Malaysia; 2 Faculty of Human Ecology, Universiti Putra Malaysia, Serdang, Selangor Darul Ehsan, Malaysia; Universidad Peruana Cayetano Heredia, Peru

## Abstract

**Background:**

We examined the relationship between morale measured by the Philadelphia Geriatric Morale Scale (PGC) and disability, social support, religiosity, and personality traits. Instruments predicting morale were then tested against PGC domains.

**Methods:**

The study utilized a cross-sectional survey with a multistage cluster sampling design. Instruments used were disability (disease burden; WHO Disability Score-II, WHODAS-II), social support (Duke Social Support Scale, DUSOCS; Lubben Social Network Scale, LSNS-6; Medical Outcomes Study Social Support Survey, MOS-SSS), religiosity (Revised Intrinsic-Extrinsic Religious Orientation Scale, I/E-R), and personality (Ten-Item Personality Inventory, TIPI). These were plotted as bar charts against PGC, resolved with one-way ANOVA and Kruskal-Wallis tests, then corrected for multiple comparisons. This process was repeated with PGC domains. Contribution of factors was modeled using population attributable risk (PAR) and odds ratios. Effect of confounders such as gender, age, and ethnicity were checked using binary logistic regression.

**Results:**

All instruments showed clear relationships with PGC, with WHODAS-II and DUSOCS performing well (ANOVA p<0.001). For PGC domains, attitude toward aging and lonely dissatisfaction trended together, while agitation did not. PAR, odds ratios, and Exp(β) were disability (WHODAS-II: 28.5%, 3.8, 2.8), social support (DUSOCS: 28.0%, 3.4, 2.2), religiosity (I/E-R: 21.6%, 3.2, 2.1), and personality (TIPI: 27.9%, 3.6, 2.4). Combined PAR was 70.9%.

**Conclusions:**

Disability, social support, religiosity, and personality strongly influence morale in the elderly. WHODAS-II and DUSOCS perform best in measuring disability and social support respectively.

## Introduction

Depression in the elderly can coexist and be easily confused with dementia and general debility due to ill health. In Malaysia, previous studies conducted in patients attending healthcare facilities have demonstrated that the prevalence of major depression in the elderly is about 13%, most of which is undiagnosed [1], [2]. Unpublished data from the authors using the Geriatric Mental State (GMS) AGECAT diagnostic system show a nationwide prevalence of about 12% sub-clinical (level 1–2) and 2% clinical (level 3–5) depression.

While proper clinical evaluation by an appropriately trained health professional is the best way to diagnose depression in the elderly, this is resource intensive and impractical for population screening. There have been a number of tools such as the Geriatric Depression Scale (GDS) developed specifically to detect depression in the elderly. In general, these use simple wordings and less complex responses, and are appropriate for older or less competent individuals [3].

The Philadelphia Geriatric Center Morale Scale (PGC) measures well-being in the elderly, which is highly correlated to depression (r = 0.75–0.85) when tested both in Western and Asian populations [3], [4], [5], [6]. The high inter-correlations suggest that the PGC can also act as a proxy marker for mood and hence depression, as has been done in some studies [7]. The PGC has also been shown to measure mood as effectively as some of the more established clinical depression scales, and in Asian populations depression is the predominant component of the PGC [5], [8].

The PGC treats morale as multidimensional, being composed of three domains: agitation, attitude toward own aging, and lonely dissatisfaction. Agitation represents general anxiety in the elderly, attitude towards own ageing captures the individual's perception of life change, while lonely dissatisfaction encompasses contentment towards the social interaction that the individual is receiving [9]. There are seventeen questions in total, answered as binary (yes-no or similar) responses. The individual domain scores can be obtained as a simple sum total of the relevant questions, and the composite score gives an overall morale rating. In general, a composite score from 13–17 represents high morale, 10–12 intermediate morale, while scores below 9 suggest low morale. The composite score from the PGC gives essentially the same information as the GDS, with similar patient profiles [3], [6].

Factors that may be associated with morale in the elderly include disability from chronic illness, and level of social support. The World Health Organization Disability Assessment Schedule II (WHODAS-II) assesses disability based on the International Classification of Functioning, Disability, and Health model [10], [11]. It has been validated cross-culturally for use in patients with chronic diseases and also in older people [12], [13]. The Duke Social Support and Stress Scale (DUSOCS), Lubben Social Network Scale (LSNS-6), and the Medical Outcomes Study Social Support Survey (MOS-SSS), are all simple measures of social support received by an individual, and are all well-accepted and validated [14], [15], [16].

Both religious orientation and personality traits can influence morale, and this effect may be more pronounced in the elderly [17], [18]. The Revised Intrinsic-Extrinsic Religious Orientation Scale (I/E-R) and the Ten-Item Personality Inventory (TIPI) are both simple and validated scales which can be used to measure these attributes [19], [20]. In general, higher scores on the I/E-R suggest greater religiosity while higher TIPI scores reflect more positive personality traits.

In this study, we examined the hypothesis that disability from chronic illness, level of social support, religious orientation, and personality traits affect morale in the elderly. We also tested the instruments selected to see which of them performed best as predictors of poor morale. Finally, we assessed the best performers of the instruments together with the I/E-R and TIPI against the three domains of morale from the PGC.

## Methods

A waiver of informed consent was obtained from the University Medical Ethics Review Board in accordance with current guidelines on Good Clinical Practice and the Declaration of Helsinki.

### Data Collection

The PGC along with the other instruments (WHODAS-II, DUSOCS, LSNS-6, MOS-SSS, I/E-R, TIPI) were administered as part of the “Patterns of Social Relationships and Psychological Well Being Among Older Persons in Peninsular Malaysia” survey conducted by trained interviewers from Universiti Putra Malaysia in 2007–2008. A small scale pilot study was performed to sort out any methodological issues prior to the full survey.

In addition, respondents were asked about the presence of chronic diseases groups which may contribute to disability: cardiovascular disease (diabetes mellitus, hypertension, hyperlipidemia, heart disease, stroke), cancer (all types), respiratory disease (asthma, obstructive airways disease, pulmonary tuberculosis), uncorrected visual or hearing impairment, renal disease, gastrointestinal disease (gastrointestinal, liver disease), musculoskeletal pain, psychiatric conditions (all types) and others (not included in list).

The survey used a multistage cluster sampling design, with clusters represented by enumeration blocks (EB) supplied by the Malaysian Department of Statistics. The whole of Malaysia was divided into small geographic units called EB for the last national census in 2000, including all residents and foreigners who intended to stay in Malaysia for at least six months during the census year. For this survey, Peninsular Malaysia was divided into four regions from which 80 EB in total were randomly selected proportional to the size of each region, urban-rural distribution, and racial composition, out of a total 52,877 EB. An additional 20 EB were added later sampling only Chinese residents after the initial survey showed a low response rate from this ethnic group as the original interviewers could not speak the local dialect. Four interviewers at a time were sent and they started at the geographic centre of each EB. The interviewers were assigned to consecutive houses to look for residents who were: above the age of 60 years, a Malaysian citizen, and willing to be interviewed. They were excluded from the study if mobility, mental functions, or hearing were sufficiently impaired to prevent them from completing the survey. Only one person from each household was interviewed, and if more than one resident in a house qualified, the person interviewed was chosen randomly. If suitable residents could not be found in a household, no replacements were sought. Each EB had a target recruitment of twenty people, but less could have been recruited if suitable residents were not found in the allocated houses.

### Scoring

The WHODAS-II in this study was scored using a simple sum scoring method, with questions rated between 0 (no problems) to 4 (very serious problems), giving an overall score range between 0–48 points for all twelve questions, which was then converted into a percentile score.

The DUSOCS was interviewer administered and scored according to standard instructions to give the overall Social Support score as a percentile. Missing values were scored as zero unless all values were missing, which then invalidated the score for that respondent [21]. The LSNS-6 was assessed using simple sum scoring with a range from 0 to 30 [22]. The MOS-SSS was condensed from a 5-point to a 4-point Likert scale but otherwise scored according to standard instructions to give a percentile score [23]. The scale was condensed following the pilot study which showed that the elderly could not differentiate between the second and third points on the scale.

The I/E-R was scored according to standard instructions into three domains: intrinsic (I), extrinsic personal (Ep), and extrinsic social (Es), which were converted to percentile scores to aid comparison [19]. The TIPI was scored into five personality domains by simple summation of the relevant item scores [20]. The scale was condensed from a 7-point to a 5-point Likert scale similar to the MOS-SSS, and the final scores multiplied by (7/5). As the domains were assessed by paired questions, missing values were replaced by the other item of the pair. Any domain with both items missing invalidated the score for that respondent. Both the I/E-R and TIPI were summed to give percentile composite Religiosity and Personality scores.

Unless specified in the original scoring instructions, missing values were replaced by the mean of the remaining values in the scale provided internal consistency was previously demonstrated to be high (Cronbach's alpha >0.7). Up to one missing value could be replaced and further missing values invalidated the score for that respondent. The scales treated in this way were the WHODAS-II, LSNS-6, MOS-SSS (up to one missing value per domain), and I/E-R (only the intrinsic domain).

### Analysis

Each of the instruments (WHODAS-II, DUSOCS, LSNS-6, MOS-SSS, I/E-R, TIPI) along with the total number of chronic diseases groups was assessed individually by plotting bar charts of mean scores against the PGC morale rating (high, intermediate, low). For the two scales with component domains (I/E-R, TIPI), the individual domains together with the composite Religiosity and Personality scores were plotted against PGC morale rating. Reliability for these two scales was assessed using Cronbach's alpha, comparing the respective component domains.

The difference in mean scores between PGC morale groups for each instrument was assessed using one-way ANOVA. Normality of the scores was checked by plotting a histogram and with the Kolmogorov-Smirnov test, while equality of variances was checked using Levene's test. Correction of the ANOVA p-value for multiple comparisons was done using Tukey's HSD if variances were equal, or with Tamhane's T2 for unequal variances. If the scores were not normally distributed, then the results would be rechecked with the non-parametric Kruskal–Wallis test.

The performance of the instruments in each category (WHODAS-II, total chronic disease groups for disability from chronic illness; DUSOCS, LSNS-6, MOS-SSS for level of social support) was assessed by examining the bar charts to look for clear discrimination between PGC morale groups. This was further verified by comparing the magnitude of the ANOVA F-scores and Kruskal–Wallis chi-square scores.

The best performers of the instruments in each category together with the I/E-R and TIPI (composite scores only) were plotted on bar charts against the three PGC morale domains. This was done to assess the pattern of variation of the morale domains with disability from chronic illness, level of social support, religiosity, and personality traits.

The top quartile of scores for each instrument corresponding to adverse PGC morale were assumed to be at risk for depression, while those in the bottom three quartiles were not. Respondents with a low PGC morale rating were considered to be at risk of depression, while those with intermediate and high ratings were not.

The contribution of each of the four factors (disability from chronic illness, level of social support, religiosity, and personality) to low morale was then modeled using population attributable risk (PAR). For each factor, respondents were classified into the following categories: low risk, low morale (NM); low risk, normal morale (NN); high risk, low morale (RM); high risk, normal morale (RN). Hence, PAR = (RM/(RM+NM))−(RN/(RN+NN)). The combined PAR for all four factors PAR(n) = 1−(1−PAR_1_)(1−PAR_2_)(1−PAR_3_)…(1−PAR_n_). The Odds Ratio for each factor was also calculated.

To check for confounders, the four factors were analyzed using binary logistic regression against a low PGC morale rating, with gender, age, and ethnicity as covariates. Model fit was assessed using the Hosmer and Lemeshow statistic, −2 log-likelihood, and overall correct classification percentage. Percentage of variance explained by the model was estimated using the Nagelkerke R-squared statistic. For the four factors, β-coefficients, Wald statistics, Wald statistic significance levels, and Exp(β) were obtained.

Sample size was calculated using the STEPS Sample Size Calculator from the World Health Organization [24]. Based on a 95% confidence level, 5% margin of error, an assumed baseline level of indicators of 50%, a design effect correction of 1.5, an expected response rate of 75%, and two age/sex groups (male & female only, no age stratification), the calculated sample size was 1537 subjects. This was rounded up to 1600 subjects, which was equivalent to 20 subjects from 80 EB.

All computations were performed using SPSS for Windows version 13.0 (SPSS Inc, Chicago, Illinois, USA) and Microsoft Excel 2007 (Microsoft Corp., Redmond, Washington, USA). Statistical tests were two-tailed and conducted at 5% level of significance.

## Results

**Figure 1:**
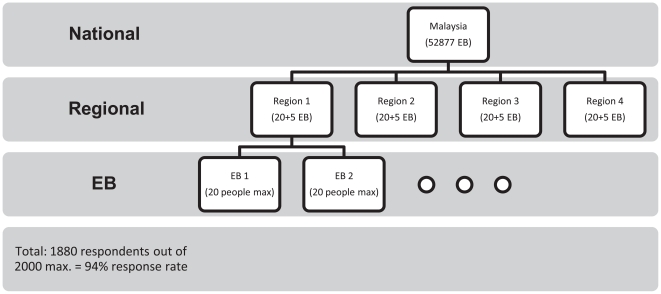
Flow chart showing recruitment process.

The sample included 1880 respondents from 100 EB in total. Basic demographic data is shown in Table 1. Based on twenty respondents from each EB, a theoretical maximum of 2000 individuals could have been recruited giving a response rate of 94%. Feedback from the interviewers suggested that most of the non-response was due to language barriers during the initial survey of 80 EB, especially with older Chinese who speak a variety of dialects. This was corrected in the follow-up survey of 20 EB in which only interviewers proficient in the local dialects were employed. The flow chart for recruitment is given in Figure 1.

**Table 1:** Basic demographic data of respondents.

Demographic Data	Distribution
Gender	47.4% males, 52.6% females
Ethnicity	74.6% Malays, 17.0% Chinese, 7.2% Indians, 1.0% Indigenous Races, 0.2% Others
Age group	54.5% in the 60–69 age group, 33.9% in the 70–79 age group, 11.6% in the over 80 age group

All respondents had valid PGC scores with a mean of 11.5 (95% CI 11.3–11.6) and a standard deviation of 3.5. The scores were not normally distributed as shown by the histogram and Kolmogorov-Smirnov test (p<0.05). The distribution was skewed towards higher scores (skew = −0.77), but not peaked (excess kurtosis = 0.03). The percentage of invalid scores for each of the instruments was: WHODAS-II (0.05%), DUSOCS (0%), LSNS-6 (0%), MOS-SSS (0.10%), I/E-R (1.8%), TIPI (0%). Overall, missing values were few enough that composite scores could still be calculated for most respondents.

**Figure 2:**
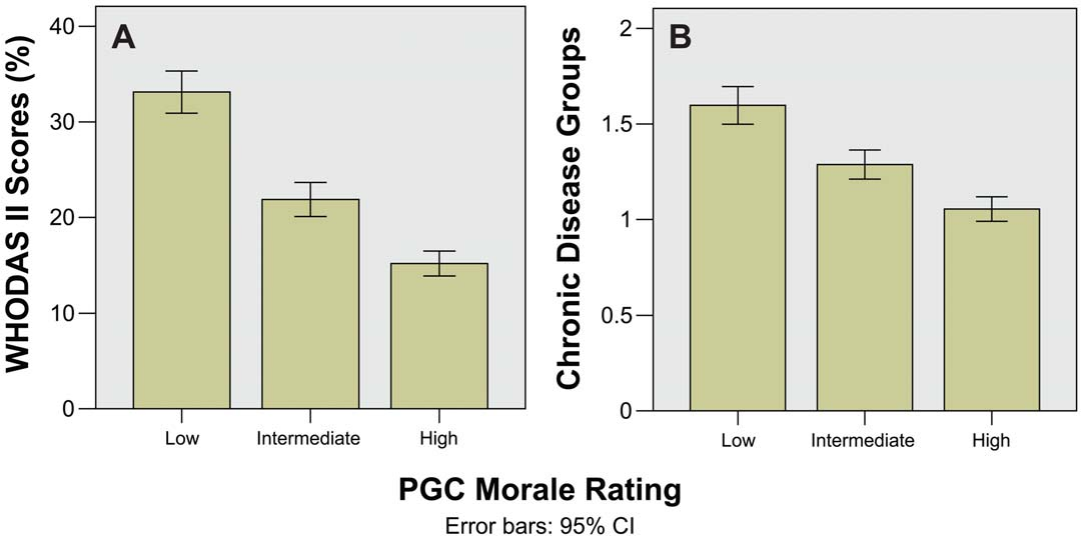
Bar charts showing chronic illness disability against PGC morale rating. A) mean WHODAS-II scores, and B) total number of chronic disease groups.

**Figure 3:**
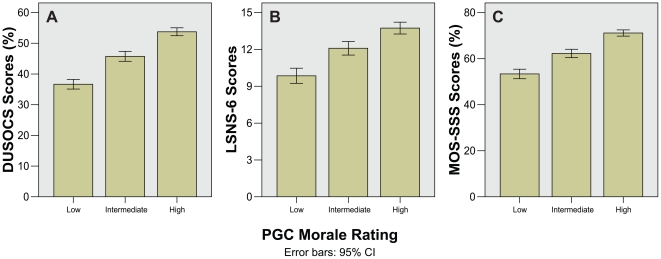
Bar charts showing social support levels against PGC morale rating. A) DUSOCS, B) LSNS-6, and C) MOS-SSS mean scores.

Bar charts for WHODAS-II and chronic disease groups show a clear trend towards worsening PGC morale rating with increasing disability from chronic illness (Figure 2A, 2B). Similarly, bar charts for DUSOCS, LSNS-6, MOS-SSS all showed that poorer social support was associated with worsening PGC morale rating (Figure 3A, 3B, 3C). These differences were reflected in the one-way ANOVA and Kruskal–Wallis tests, which remained highly significant (p<0.001) even after correction for multiple comparisons (Table 2).

**Table 2:** Comparison between instruments measuring disability from chronic illness, level of social support, religiosity, and personality.

Instrument	Normally Distributed (K-S* & Histogram)	Equal Variances (Levene's Test)	ANOVA F-Score	Kruskal–Wallis Chi-Square	Tukey's HSD** Tamhane's T2
WHODAS-II	No	No	109.0	233.4	<0.001
Chronic Disease	No	No	47.4	94.7	<0.001
DUSOCS	No	Yes	131.5	228.4	<0.001
LSNS-6	No	No	48.4	89.6	<0.001
MOS-SSS	No	No	110.3	191.0	<0.001
I/E-R I	No	No	27.2	61.9	0.036
I/E-R Es	No	Yes	29.5	55.1	0.084
I/E-R Ep	No	No	27.5	62.5	0.047
I/E-R Composite	No	No	52.8	104.8	0.003
TIPI Extraversion	No	Yes	86.7	154.3	<0.001
TIPI Agreeableness	No	No	37.1	74.1	0.003
TIPI Conscientiousness	No	Yes	37.2	72.3	0.031
TIPI Emotional Stability	No	No	185.3	318.9	<0.001
TIPI Openness	No	No	10.0	22.8	0.688
TIPI Composite	No	No	161.9	277.2	<0.001

*K-S = Kolmogorov-Smirnov test for normality.

**test used for correction of p-value for multiple comparisons depended on equality of variances.

**Figure 4:**
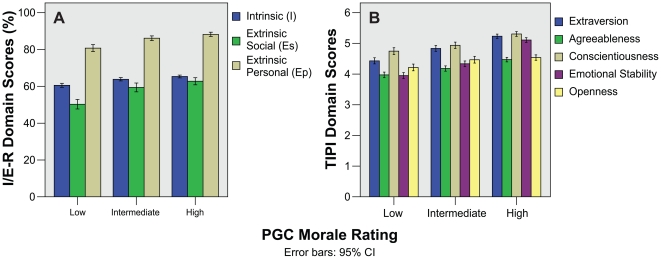
Bar charts showing Religiosity and Personality domain scores against PGC morale rating. A) I/E-R Religiosity, and B) TIPI Personality mean domain scores.

**Figure 5:**
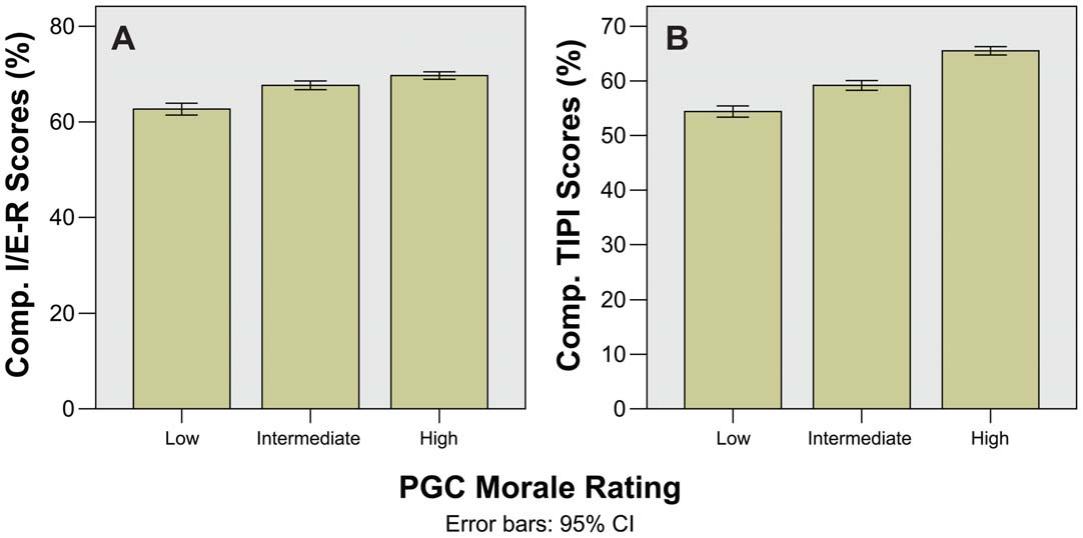
Bar charts showing composite Religiosity and Personality scores against PGC morale rating. A) I/E-R Religiosity, and B) TIPI Personality mean composite scores.

Bar charts for I/E-R Religiosity and TIPI Personality domain scores all show higher PGC morale rating with increasing religiosity and positive personality traits (Figure 4A, 4B). This was also seen in the respective composite scores which showed clear differentiation between PGC morale rating groups (Figure 5A, 5B). The one-way ANOVA and Kruskal–Wallis tests showed significant differences in most domain scores except for the I/E-R Extrinsic Social (Es) and TIPI Openness to Experiences domains after correction for multiple comparisons (Table 2). The composite I/E-R Religiosity and TIPI Personality scores showed highly significant differences even after correction for multiple comparisons (p = 0.003 and p<0.001 respectively). Reliability analysis for I/E-R and TIPI gave alpha values of 0.52 and 0.58 respectively.

Of the instruments measuring disability from chronic illness, the WHODAS-II showed the greatest differentiation between PGC morale rating groups on the bar charts and had the highest ANOVA F-scores and Kruskal–Wallis chi-square scores. For the instruments measuring the level of social support, the DUSOCS showed the clearest differentiation between groups.

**Figure 6:**
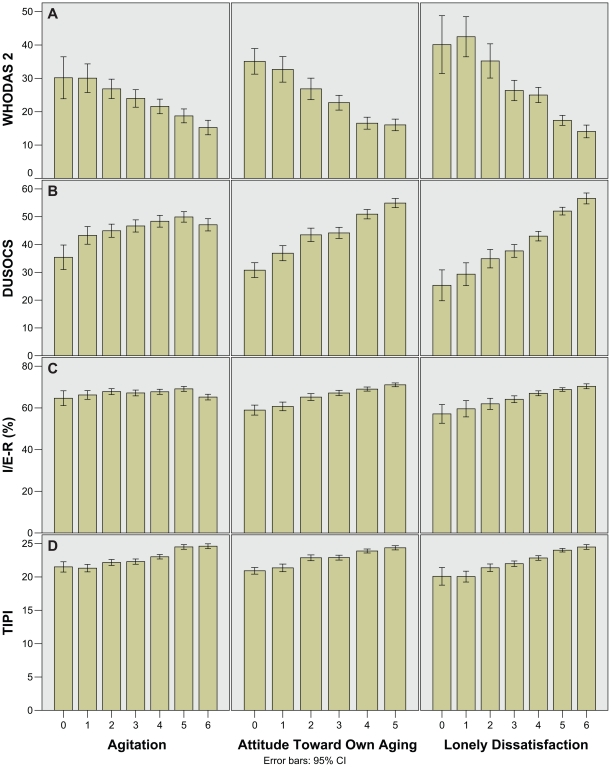
Bar charts showing WHODAS-II, DUSOCS, I/E-R, and TIPI against PGC morale domains. A) WHODAS-II, B) DUSOCS, C) I/E-R, and D) TIPI mean scores.

Bar charts of WHODAS-II, DUSOCS, I/E-R, and TIPI against the three PGC morale domains are shown in Figure 6A–D respectively. Greater disability from chronic illness (higher WHODAS-II scores) was associated with a consistent fall in all three morale domains. Reduced social support (lower DUSOCS scores) was associated with a poorer attitude towards ageing and more lonely dissatisfaction, but increased anxiety only at very low levels of support. Increased religiosity somewhat improved attitude towards ageing and satisfaction, but made no difference to anxiety. Positive personality traits were associated with a modest but consistent improvement in all morale domains.

The PAR for each of the four factors was as follows: WHODAS-II (28.5%), DUSOCS (28.0%), I/E-R (21.6%), and TIPI (27.9%). The combined PAR for all four factors was 70.9%. The Odds Ratios respectively were: WHODAS-II (3.8), DUSOCS (3.4), I/E-R (3.2), and TIPI (3.6).

Binary logistic regression showed that model fit was good with a non-significant Hosmer and Lemeshow statistic (p = 0.476), −2 log-likelihood of 1741.5, and a 78.8% overall correct classification percentage. The Nagelkerke R-squared statistic was 0.249. The relevant statistics for all factors are shown in Table 3.

**Table 3:** Relevant statistics for the four factors from logistic regression analysis.

Factors	β	Wald	Sig.	Exp(β)
WHODAS-II	1.034	67.827	<0.001	2.813
DUSOCS	0.770	36.444	<0.001	2.159
I/E-R Composite	0.756	22.677	<0.001	2.129
TIPI Composite	0.882	50.044	<0.001	2.416

## Discussion

From the results, we can confirm that disability from chronic illness, level of social support, religious orientation, and personality traits all have a strong influence on morale in the elderly. While the study looked specifically at morale, the high correlation of PGC scores with mood suggests that a low morale puts an individual at risk for depression [3], [6].

The effect of selected confounders such as gender, age, and ethnicity, was adjusted for by including them as covariates in binary logistic regression. The Exp(β) statistic is essentially the same as an odds ratio, but taking into account the effect of interactions between factors and with the confounders. The results for the four factors mirror closely the calculated odds ratios, and are still highly significant (Table 3).

All of the instruments examined were adequate measures of disability from chronic illness and level of social support, in relation to morale in the elderly. Of these, the WHODAS-II and DUSOCS respectively had the best performance in differentiating PGC morale groups, and furthermore are simple to complete and do not require equipment or specially trained staff. As both disability and social support are potentially modifiable, it makes sense to look for these factors when screening for low morale. In contrast, religiosity and personality are difficult to modify and screening for these is normally useful only for research. This was taken into account in the study design with more attention paid to the modifiable risk factors.

In the original instructions, the I/E-R, and TIPI are not meant to be summed into composite scores. However, during analysis we noticed that all the component domains for both instruments trended together (Figure 4A, 4B), and felt that using simple sum composite scores would add value to the study. This was borne out when both composite scores were able to clearly differentiate PGC morale rating groups, with highly significant ANOVA and Kruskal–Wallis scores, with large PAR values. While alpha reliability values were less than ideal (0.52 and 0.58 for I/E-R and TIPI respectively), this was expected as the individual domains measure different aspects of religiosity and personality.

Analysis of the PGC component domains showed that attitude toward own aging and lonely dissatisfaction trended together, while agitation did not. One possible reason for this could be that agitation is internally directed while attitude and satisfaction are externally directed. This is reflected in the religiosity bar charts (Figure 6C) where attitude and satisfaction (external) respond to increasing religiosity but agitation (internal) does not. Similarly, agitation did not relate well to the level of social support unless it was very low (Figure 6B).

The two most important personality traits affecting PGC morale are Extraversion and Emotional Stability (Table 2). This is intuitive as individuals who are emotionally stable are less likely to have low morale, and extroverts by their nature are more cheerful and likely to seek out social support.

Finally, this study has a number of weaknesses which may impair its generalizability to the elderly population. The PGC while being a good screening tool for morale should have been paired together with another measure of mood such as the GDS. This would have allowed us to confirm that the findings were related to depression rather than being a peculiarity of the PGC, although this is mitigated by the fact that the PGC is a good proxy marker of depression [3], [4], [5], [6], [7], [8].

There was a systematic bias against severe disability due to the sampling method which excluded people who were too disabled to complete the survey. However, there was no good way of working around this issue as the alternative would be to rely on secondary information from caregivers, which would be vulnerable to confirmation bias. While the PGC utilizes simple wording and is suitable for administration in older people, severe hearing or cognitive impairment makes it difficult to use with confidence, and some studies using the PGC have excluded subjects in this category [4].

It would have been desirable to use the same Likert scale as the original instruments for the MOS-SSS and TIPI, so that the results can be directly compared with previously published work. However, from the feedback given by respondents during the pilot study, it was judged that the additional information obtained would have been unreliable due to the limited comprehension of some of the elderly, hence the modified scales used.

In spite of these weaknesses, the generalizability and external validity of the study results are good as the number of respondents was large, response rate was high, and the percentage of invalid instrument scores was low. Much care was taken to account for ethnic bias by resampling groups with a low response rate such that the overall racial composition was a good approximation for the ethnic mix in Peninsular Malaysia. Finally, most of the differences found were highly significant and it is likely that these findings would persist across different populations.

### Implications

For physicians involved in clinical management of older persons, the results of this study suggest that screening for low morale and occult depression in patients with significant disability or poor social support is highly advisable. This can be carried out with minimal impact on consultation time using a purpose designed instrument such as the PGC or GDS, which can even be administered informally in a clinic waiting area.

Reducing disability from chronic disease and improving social support can potentially alleviate low morale without resorting to psychiatric medications, most of which have significant adverse effects in the elderly. This can be done through proper management of the underlying disease, adequate symptom relief, rehabilitation, and referral to social services. While religiosity and personality traits are difficult to modify, where circumstances and resources permit, these factors can also be looked at. There are numerous faith based programs which can act as adjuncts to standard medical therapy, and cognitive behavioral therapy may be useful in correcting overtly dysfunctional personality traits.

### Conclusions

From this study, we can conclude that disability from chronic illness, level of social support, religious orientation, and personality traits strongly influence morale in the elderly. Furthermore, WHODAS-II and DUSOCS perform best in assessing disability from chronic illness and level of social support respectively, and should be included when screening for low morale and depression in the elderly.
